# Neuroscience and Values: A Case Study Illustrating Developments in Policy, Training and Research in the UK and Internationally**

**DOI:** 10.4103/0973-1229.77428

**Published:** 2011

**Authors:** K. W. M Fulford

**Affiliations:** * *Dphil, FRCP, FRCPsych. Fellow of St Cross College, Member of the Philosophy Faculty and Honorary Consultant Psychiatrist, University of Oxford; Emeritus Professor of Philosophy and Mental Health, University of Warwick; Editor, Philosophy, Psychiatry, and Psychology; and Special Advisor for Values-Based Practice, Department of Health, London*; ** *Revised and peer reviewed version of a Plenary Session Paper for an International Seminar on Mind, Brain, and Consciousness, Thane College Campus, Thane, India, January 13-15, 2010*

**Keywords:** *Evidence-based practice*, *Values-based practice*, *Diagnostic and Statistical Manual*, *Diagnosis*, *Classification*, *Psychosis*, *Schizophrenia*, *Spiritual experience*, *Religious experience*

## Abstract

In the current climate of dramatic advances in the neurosciences, it has been widely assumed that the diagnosis of mental disorder is a matter exclusively for value-free science. Starting from a detailed case history, this paper describes how, to the contrary, values come into the diagnosis of mental disorders, directly through the criteria at the heart of psychiatry’s most scientifically grounded classification, the American Psychiatric Association’s DSM (Diagnostic and Statistical Manual). Various possible interpretations of the prominence of values in psychiatric diagnosis are outlined. Drawing on work in the Oxford analytic tradition of philosophy, it is shown that, properly understood, the prominence of psychiatric diagnostic values reflects the necessary engagement of psychiatry with the diversity of individual human values. This interpretation opens up psychiatric diagnostic assessment to the resources of a new skills-based approach to working with complex and conflicting values (also derived from analytic philosophy) called ‘values-based practice.’ Developments in values-based practice in training, policy and research in mental health are briefly outlined. The paper concludes with an indication of how the integration of values-based with evidence-based approaches provides the basis for psychiatric practice in the twenty-first century that is both science-based and person-centred.

## Introduction

A significant practical spin-off from the recent upsurge of cross-disciplinary work between philosophy and psychiatry (Fulford *et al*., 2003) has been the development of a new skills-based approach to working with complex and conflicting values called values-based practice (Woodbridge and Fulford, 2004).

That there should be a need for *values*-based practice in psychiatry‘s ’decade of the brain’ came to many at best as something of a surprise, at worst, with its implications for a more equal role for service users alongside clinicians and researchers, as a positive threat to the emerging sciences of the field (Spitzer, 2005). Yet, as I will outline in this paper, values and science, at least as reflected in psychiatric diagnostic classifications, are neither incidental nor inimical to each other, but twin and complementary components of a psychiatry that as a medical discipline is both science-based and person-centred.

## Case Study: Simon

Simon (40) was a senior, black, American lawyer from a middle-class, Baptist family. Before the onset of his symptoms, he reported sporadic, relatively unremarkable, psychic experiences.

Around four years before the first interview, his hitherto successful career was threatened by legal action from his colleagues. Although he claimed to be innocent, mounting a defence would be expensive and hazardous. He responded to this crisis by praying at a small altar that he set up in his front room. After an emotional evening’s ‘outpouring,’ he discovered that the candle wax had left a ‘seal’ (or ‘sun’) on several consecutive pages of his bible, covering certain letters and words. He described his experiences thus:

“I got up and I saw the seal that was in my father’s bible and I called X and I said, you know, ‘something remarkable is going on over here.’ I think the beauty of it was the specificity by which the sun burned through. It was … in my mind, a clever play on words.”

Although the marked words and letters had no explicit meaning for anyone from his own Baptist background, Simon interpreted this, and a series of subsequent similar events, as a direct communication from God which signified that he had a special purpose or mission as “ … *the living son of David … and I’m also a relative of Ishmael, and … captain of the guard of Israel*”.

He expressed these beliefs with full conviction and when confronted with scepticism, commented, “*I don’t get upset, because I know within myself, what I know*”.

### Simon in the International Classification of Diseases

Simon’s story was first published by the British psychologist, Mike Jackson (1997), in a study of delusion and spiritual experience. Simon, it would seem, had a delusional perception (the revelations in his ‘suns’ or wax seals) which in the ICD (International Classification of Diseases, World Health Organization, 1992) suggests a diagnosis of schizophrenia or other psychotic illness. Yet, Simon (along with many others in Jackson’s study) was much *empowered* by his experiences; he won his court case; went on to become a successful lawyer and always considered his experiences to be deeply spiritual in nature.

### Simon in the Diagnostic and Statistical Manual

If Simon’s own understanding of his experiences is in conflict with the ICD, it is consistent with the DSM (the Diagnostic and Statistical Manual, American Psychiatric Association, 1994). Thus, the DSM includes, in addition to traditional symptom-based diagnostic criteria, what it calls ‘criteria of clinical significance.’ For a diagnosis of schizophrenia in the DSM, therefore, Simon would have to show not only the relevant symptoms (to satisfy Criterion A), but also a *deterioration* in social and/or occupation functioning (to satisfy Criterion B, the relevant criterion of clinical significance (p285). To the extent, then, that Simon’s occupational functioning was actually *enhanced* by his experiences, he *failed* to satisfy Criterion B and hence did not, despite satisfying Criterion A (the symptom-based criterion), have schizophrenia at all according to the DSM, but a spiritual experience.

### Facts and values in the Diagnostic and Statistical Manual

It might be thought that the success of the DSM in providing a more face-valid interpretation of Simon’s experiences than the ICD reflects its more explicit evidence-base (American Psychiatric Association, 1994, p XV). Closer inspection, however, suggests to the contrary that the key to the DSM interpretation is its more explicit *values*-base. Consider Criterion B: it requires both a *change* in functioning (a matter of fact) and a change for the *worse* (which is a matter of values).

DSM thus makes explicit what many have argued is implicit in psychiatric diagnostic concepts generally, namely that they are deeply value laden (Fulford, 1989; Sadler, 2005). The question that arises, then, is what interpretation should be placed on this.

### Interpretations various

The value-laden nature of psychiatric diagnostic concepts is capable of a number of different interpretations (Fulford, 1989). In the psychiatry/antipsychiatry debates of the 1960s and 1970s, for example, psychiatrists such as R.E. Kendell (1975), [and Singh and Singh recently (2009)], argued that the relevant values would disappear as future scientific advances established biological correlates of psychological abnormalities, whereas so-called antipsychiatrists, by contrast, claimed that these same values showed mental disorders to be irreducibly and regardless of future scientific advances, a matter of ‘morals not medicine’ (Szasz, 1960); and in the now very large literature on concepts of disorder, there have been many subsequent attempts to find a middle ground between what I have called elsewhere the ‘values in *versus* values out’ extremes of these early positions by delineating, in one way or another, what is taken to be a value-free element of their meanings (Fulford, 2000).

I do not have space here to do justice to these different interpretations (but see Fulford, 1989, chapter 1). The issues raised by the psychiatry/antipsychiatry debate are important however, not just theoretically but because they go to the heart of diagnosis and treatment in the *practice* of psychiatry, and I return to these issues below. First, however, I want to introduce briefly a very different way of understanding the prominence of values in psychiatric diagnosis that we owe to the work of R M Hare and others in the Oxford ‘school’ of analytic philosophy. As we will see, this work, although originally abstract and theoretical in nature, directly underpins the practical tools of values-based practice.

### Hare on values

As a former White’s Professor of Moral Philosophy in Oxford, R M Hare (Hare, 1952, 1963) worked with J L Austin (Austin, 1956-7), Geoffrey Warnock (Warnock, 1971), J O Urmson (Urmson, 1950) and others in what is called analytic ethics. What this means is that rather than focusing on substantive moral questions (‘*is* this or that action right or wrong?’), this group of philosophers focused on the logic (the meanings and implications) of value terms (‘what does it *mean* to say that this or that action is right or wrong?’).

This all sounds rather abstract and indeed like mathematics analytic philosophy *is* abstract. Yet, also like mathematics, it is the abstract nature of analytic ethics that makes it a powerful approach when combined with an empirical discipline like psychiatry (think here of the power of mathematics when combined with observational physics). In the present case, there is a key point that we can take particularly from Hare’s work that explains the more value-laden psychiatric diagnosis and thereby provides the theoretical grounding for the applied tools of values-based practice.

### Values visible

**Figure 1:**
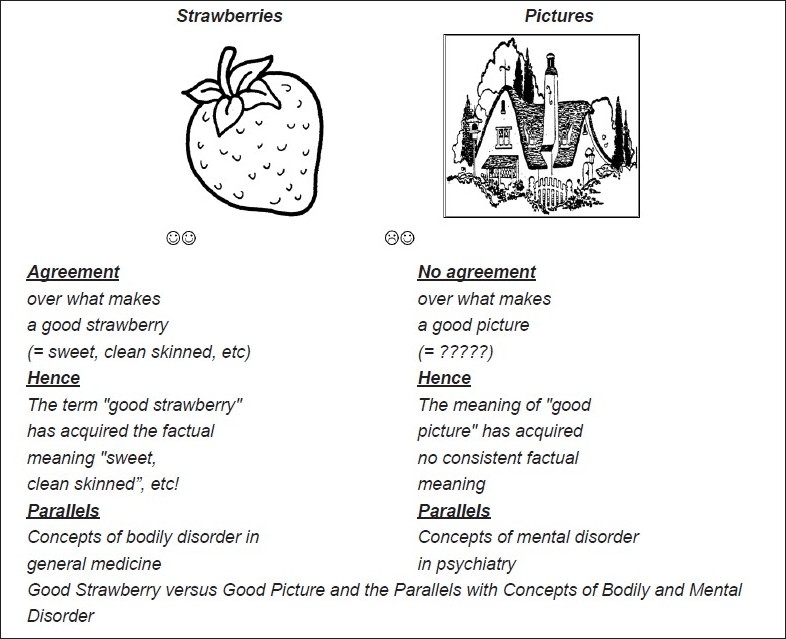
Good Strawberry and Good Picture

The key point to take from Hare’s work in the present instance can be summed up in the slogan ‘*visible* values = *diverse* values.’ The essence of Hare’s argument, as the Figure illustrates [Figure 1], is that *all* value term, including such generic value terms as ‘good’ and ‘bad,’ may come to appear value-free and thus more like factual terms, where the values they express are largely agreed or settled upon.

Hare’s example, shown in the left-hand side of the Figure, was of ‘good strawberry,’ this being a use of the value term ‘good’ in which it carries largely factual meaning, namely that the strawberry in question is red, sweet and grub-free, etc. This is essentially because most people have shared values when it comes to strawberries, that is, that strawberries should be red, sweet and grub-free, etc., and hence, this *factual* meaning has become attached by association to the use of the value term ‘good strawberry.’ Conversely, ‘good’ used of pictures, as in the right-hand side of the Figure, over which people’s values vary widely, has no shared factual meaning and thus retains overtly evaluative connotations.

### Visible values in psychiatric diagnostic concepts

Returning now to the medical concepts, an exactly parallel argument explains the relatively value-laden nature of psychiatric diagnostic concepts compared with concepts of bodily disorder. Again, this is illustrated by the Figure 1. In a direct parallel with Hare’s argument about value terms in general, we see that psychiatric diagnostic concepts are relatively value-laden, neither because psychiatry is a matter of ‘morals not medicine’ (as the anti-psychiatrists suggested, as above), nor because of the supposed underdevelopment of psychiatric science (as Kendell and others believed), but because the values involved in diagnostic judgments in psychiatry, in contrast with the corresponding judgments in bodily medicine, are *particularly diverse* (Fulford, 1989, chapter 5).

Thus, the criteria for good and bad heart functioning, for example, paralleling ‘good strawberries,’ are largely settled and agreed upon, and this is true by and large of all the areas with which (acute) bodily medicine is primarily concerned. By contrast, however, the areas with which psychiatry is primarily concerned–emotion, desire, belief, motivation, sexuality and so forth–are all areas in which our values, paralleling ‘good pictures,’ are highly diverse.

### Values-based practice: The theory

Again, I do not have space here to go into the arguments for and against this interpretation in detail. Nonetheless, it is important to note a number of theoretical points to the extent that these bear on the issues for diagnosis and treatment in psychiatry raised by the debate about mental illness (as outlined above).

### The is-ought debate in philosophy

Like all research-led disciplines, the logic of values remains an area of on-going debate and Hare’s analysis is far from being universally accepted. Indeed, it falls within a 200-year debate in philosophy about the logical relationship between factual and evaluative meaning (the so-called ‘is-ought’ debate), a debate that continues to this day (see for example, Putnam, 2002).

### Abnormality

The issues in the ‘is-ought’ debate in philosophy are reflected in those raised in the psychiatry/antipsychiatry debate about the concept of mental illness (noted above) and in the modern extension of that debate to concepts of disorder in general (Fulford, 2003). The key issue for practice is whether *abnormality* of functioning, whether in psychiatry or indeed in any other area of medicine, although ostensibly a value-laden notion can be defined (at least in part) value-free (Fulford, 1989 and 2000).

We can see the importance of the concept of abnormality in the central role that Criterion B, as a criterion of social/occupational *dys*function, played in the differential diagnosis in Simon case. Whether we took Simon to be suffering from a psychotic disorder or going through a religious experience turned, in the DSM, on deciding not just whether his social/occupational functioning had changed (this being a matter of fact) but had changed *for the worse* (this being a matter of values). So applied to Criterion B, and by extension to the wider problem of the nature of abnormal psychological (or any other) functioning, the debate about concepts of disorder becomes a debate about whether the requisite value judgments can be replaced by (or redefined in terms of) one or more facts.

### Values-in *vs* values-out in diagnosis and treatment

This is where different interpretations again become relevant. One view (the view that I characterised elsewhere as the ‘values-out’ view, Fulford, 2000) is that we should either exclude values or at the very least work toward a consensus as the basis of consistent diagnostic criteria and treatment guidelines. This is a common sense view with many credible advocates (such as R. E. Kendell, no less, as above) and again, I cannot do full justice to the many variations on the values-out approach here (but see Fulford, 2003).

The values-out approach, however, whatever its merits, remains promissory on it, proving possible to exclude values. This is proving difficult in practice (witness the prominence of values in the DSM; Sadler, 2005) and may be impossible in principle (Fulford, 1998). It is also not risk free; consensus on all values too easily slides to *enforced* consensus with all its inherent risks of the abusive practices to which psychiatry has been so peculiarly subject throughout its history (Fulford *et al*., 2003).

The alternative ‘values-in’ approach is to accept that diversity of human values is indeed part of the clinical challenge faced by psychiatry and to work with rather than against it. This is what values-based practice as described further below seeks to do.

### Values-in *vs* values-out in science

A further reason for seeking a values-out solution to the problem of dealing with the diversity of human values in psychiatric diagnosis is the belief that these are somehow prejudicial to the hard science agenda of establishing the biological correlates of psychological abnormality. Again, this is a concern with credible advocates and merits fuller treatment than I can give to it here.

Values-based practice, however, whatever may be true of its alternatives, is very much a partner to science in medicine. At the level of diagnosis and treatment values-based practice provides a key link in clinical decision-making between on one hand, the generalisable knowledge derived from science and reflected in evidence-based practice, and on the other hand, what evidence-based practitioners themselves have identified as the ‘unique values’ of individual patients (Sackett *et al*., 2000, page 1).

At the level of research, it clarifies the relevant research questions. Simon’s story again makes the point. There are clear research questions to be answered about the different causal processes involved in producing what we might call well-functioning psychotic experiences (as in Simon’s case) as distinct from the more familiar badly functioning psychotic experiences (as in the case of disorders such as schizophrenia). Delineating the different (presumably very different) causal pathways involved is important not only theoretically but also as the basis of developing more effective treatments. But the research required to delineate these two causal pathways has to start, like comparable research in any other area of medicine, from clear clinical differentiation of the phenomena in question. This in turn requires good clinical skills. And good clinical skills, as we will see in the next section, are the foundation of values-based practice.

### Values-based practice

Values-based practice is one of a growing number of new disciplines contributing to more effective ways of working with values in medicine (Fulford, 2004). A distinctive feature of values-based practice, and one that makes it particularly relevant to tackling problems involving diversity of values, is that it starts from and celebrates the importance of the uniqueness of the individual. Unlike ethics, therefore, which seeks to determine ‘right outcomes,’ values-based practice relies on ‘good process,’ in particular good clinical skills, as the basis of balanced decision-making where values conflict.

There are ten key elements of the process of values-based practice (Fulford and Woodbridge, 2004). As just noted, values-based practice is primarily skills-based (see below). But also important are (1) an appropriate service model (values-based practice depends on a person-centred and multidisciplinary service structure), (2) strong links between values-based and evidence-based approaches and (3) partnership between service users and service providers in clinical decision-making. A full account of these ten elements, together with a detailed case history of ‘The Artist who Couldn’t See Colours,’ is given in my (Fulford, 2004) Ten Principles of Values-Based Medicine. Further case studies illustrating the applications of values-based practice in other areas of medicine besides psychiatry are given in the launch volume for a new series from Cambridge University Press on values in medicine, *Essential Values-based Practice: Linking Science with People* (Fulford, Carroll and Peile, forthcoming).

### The development of values-based practice in mental health

As I describe in detail elsewhere (Fulford, 2008), values-based practice has been actively developed in mental health through a number of initiatives involving national and international partners representing its three key stakeholder groups–patients, professionals and policy makers–and there are now substantial resources to support values-based policy, training and service development.

The first training manual for values-based practice, called ‘Whose Values?’ (Woodbridge and Fulford, 2004), which was developed and piloted with frontline mental health staff, was launched by the UK Minister of State in the Department of Health, Rosie Winterton, at a conference in London in 2005. ‘Whose Values?’ is built around a series of training exercises in the following four skills areas of values-based practice:

1. Increased *awareness* of values and of the often surprising *diversity* of values–this is the foundation and starting point for effective values-based practice
2. The ability to reason about values using a variety of different methods (principles reasoning, utilitarianism, deontology, etc)
3. Knowledge of values derived using not only standard empirical methods (Petrova *et al*., forthcoming), but also other sources: personal narratives of patients and family carers, and a number of powerful philosophical methods, including phenomenology, hermeneutics and discursive philosophy (Fulford *et al*., 2003).
4. Enhanced communication skills–vital not only for exploring values and differences of values, but also in such areas as conflict resolution and shared decision-
5. making (Hope *et al*., 1996).

Values-based practice has been combined with policy and service development in two recent U.K. Department of Health initiatives. The first is a training package developed to support implementation of a revised Mental Health Act [Care Services Improvement Partnership (CSIP) and the National Institute for Mental Health in England (NIMHE), 2008]. The second example, which takes us back directly to the diversity of values at the heart of the problems, illustrated by the Story of Simon, is a wide-ranging consultation on best practice in assessment in mental health [National Institute for Mental Health in England (NIMHE) and the Care Services Improvement Partnership, 2008]. Consistently with the principles of values-based practice, service users and practitioners in this consultation all agreed that assessment should be (1) strengths-based (covering individual strengths and aspirations as well as needs and difficulties), (2) multidisciplinary (drawing where possible on more than one disciplinary perspective) and (3) participatory (involving a shared ‘project of understanding’ between service user and practitioner).

## Conclusions [see also Figure 2]

**Figure 2:**
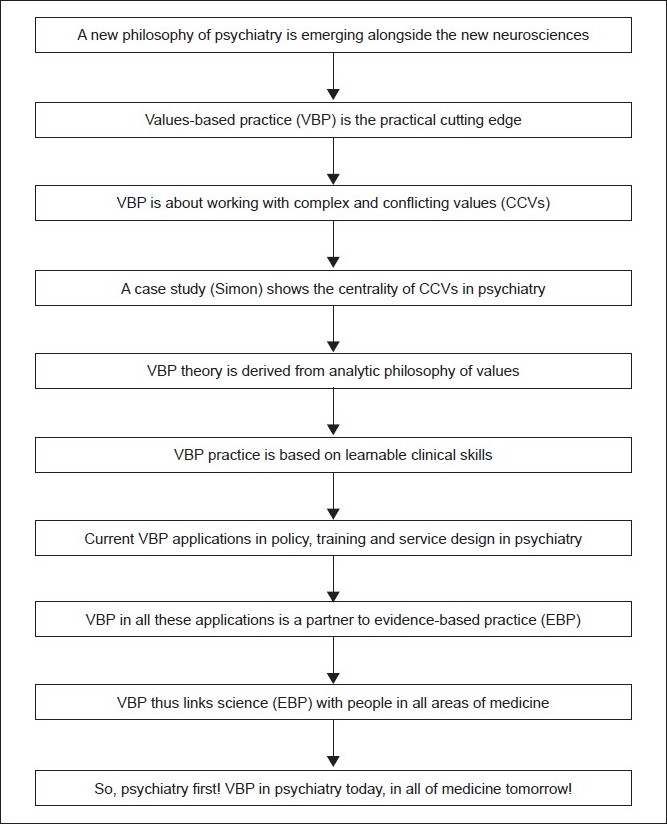
Flowchart of paper

Starting with the story of a real person, Simon, this paper has drawn on analytic work from the ‘Oxford school’ on the logic of value terms, to show that the prominence of values in psychiatric diagnostic categories is a reflection, neither of mental disorders being a matter of ‘morals not medicine’ (as the antipsychiatrists claimed), nor of psychiatry’s (supposed) underdevelopment as a science (as some of its supporters have argued), but rather of the essential engagement of psychiatry with the full diversity of individual human values.

It is important to be clear that, in terms of the slogan noted earlier, this is a ‘values *in*’ rather than a ‘facts *out*’ conclusion. What it implies, that is to say, is that good practice in psychiatry depends on pulling together-integrating, if you will–science and values, evidence-based and values-based approaches.

The need for this arises in part from the fact that, as noted earlier, psychiatry is concerned with areas of human experience and behaviour where, in addition to the relevant *evidence* being complex and conflicting (driving the requirement for evidence-based practice), the relevant *values* are also complex and conflicting (driving the requirement for values-based practice). But the need for an integrated approach also arises from the fact that, as the American neuroscientist and psychiatrist, Nancy Andreasen (2001) has argued, psychiatry is unique among medical disciplines in being irreducibly concerned, not with bodily or even mental subparts and systems, but with whole persons. And persons, as the Oxford philosopher, P. F. Strawson (1997, p 102) graphically put it, are ’… a type of entity such that *both* predicates ascribing states of consciousness and predicates ascribing corporeal characteristics … are equally applicable….’ Is it too fanciful to believe that something similar may be true of science and values?

### Take home message

Neuroscience and values need to be integrated into psychiatry as a uniquely person-centred branch of scientific medicine through the complementary processes of evidence-based and values-based practice.

## Questions That This Paper Raises

1. What are values? Are they just ethical values or do they extend to needs, wishes, etc?
2. How do values and evidence come together in medical decision-making? Can you think of *any* decision (not just in medicine) that does not depend on combining evidence and values in some way?
3. Do values come into diagnostic decisions in areas of medicine other than psychiatry? If so, why are they more explicit in psychiatry?
4. If psychiatry needs values-based as well as evidence-based processes today (reflecting the complexity of the values as well as evidence with which it is concerned clinically), do you think other areas of medicine are likely to have the same requirement (i.e., for values-based as well as evidence-based processes) in the future? (Think about the way in which science and technology are already opening up new choices, and hence more complex values, for patients, families and clinicians, for example in reproductive medicine.)
5. Given that Simon’s story is far from unique (Jackson, 1997), how might brain imaging research contribute to understanding why psychotic experiences prove so helpful to some people (who like Simon thus function as well or better than before) while to other people they are profoundly damaging (as in cases of schizophrenia)?
6. What is the effect of misdiagnosis in cases like Simon’s? Anecdotally, there is evidence that psychiatrists tend to neglect Criterion B when they have clear evidence of psychotic experiences (i.e., satisfying Criterion A in DSM). Simon avoided contact with a psychiatrist. But how might his life have gone if he had been (mis)diagnosed with schizophrenia and treated with neuroleptics?

## About the Author

KWM (Bill) Fulford is a Fellow of St Cross College, a Member of the Faculty of Philosophy and an Honorary Consultant Psychiatrist, University of Oxford; he is also Emeritus Professor of Philosophy and Mental Health in the University of Warwick Medical School; and Special Adviser for Values-Based Practice in the National Mental Health Development Unit, Department of Health, London. He is the Founder Editor of Philosophy, Psychiatry, and Psychology, and of a book series from Oxford University Press on International Perspectives in Philosophy and Psychiatry. He has published widely in the philosophy of psychiatry, including the Oxford Textbook of Philosophy and Psychiatry.
